# Theories of God: Explanatory coherence in religious cognition

**DOI:** 10.1371/journal.pone.0209758

**Published:** 2018-12-26

**Authors:** Andrew Shtulman, Max Rattner

**Affiliations:** Department of Psychology, Occidental College, Los Angeles, California, United States of America; University of Exeter, UNITED KINGDOM

## Abstract

Representations of God in art, literature, and discourse range from the highly anthropomorphic to the highly abstract. The present study explored whether people who endorse anthropomorphic God concepts hold different religious beliefs and engage in different religious practices than those who endorse abstract concepts. Adults of various religious affiliations (*n* = 275) completed a questionnaire that probed their beliefs about God, angels, Satan, Heaven, Hell, cosmogenesis, anthropogenesis, human suffering, and human misdeeds, as well as their experiences regarding prayer, worship, and religious development. Responses to the questionnaire were analyzed by how strongly participants anthropomorphized God in a property-attribution task. Overall, the more participants anthropomorphized God, the more concretely they interpreted religious ideas, importing their understanding of human affairs into their understanding of divine affairs. These findings suggest not only that individuals vary greatly in how they interpret the same religious ideas but also that those interpretations cohere along a concrete-to-abstract dimension, anchored on the concrete side by our everyday notions of people.

## Introduction

Most people in most cultures believe in the existence of supernatural beings and other supernatural concepts [1–2]. Belief in the supernatural is particularly prevalent in the United States, where nine out of ten individuals believe in God, eight out of ten believe in Heaven and angels, and seven out of ten believe in Hell and Satan [3]. Belief in supernatural beings has been studied extensively from a social perspective, where such beliefs serve to regulate social exchange and ensure intra-group cooperation [4], but less is known about the conceptual representations that underpin them.

Scholars of religious cognition generally agree that supernatural concepts are represented using the same cognitive resources used to represent natural concepts, but it is unclear how this process yields culturally-authentic concepts that are endorsed widely and strongly [5]. Indeed, from a psychological point of view, supernatural concepts present a significant challenge to standard, constructivist models of knowledge acquisition [6–7], as these models assume knowledge is acquired through direct observation and exploration of the physical world. Knowledge of supernatural beings is rarely acquired in this manner. Rather, most individuals learn about such beings from the art, literature, and discourse of their culture. How individuals make sense of this information is the focus of the present investigation. We explore differences in the interpretation of religious ideas, and how those differences relate to differences in the core concept underlying those ideas: God.

Interpreting information about God is by no means trivial. Representations of God in art, literature, and discourse range from the highly anthropomorphic (“heavenly father,” “divine ruler,” “intelligent designer”) to the highly abstract (“first cause,” “unmoved mover,” “universal spirit”). Collectively, they paint a picture of God that is neither consistent nor coherent. For example, God is said to listen to prayers, yet God is also said to be all-knowing, and an all-knowing being would be informed of the content of those prayers already. God is said to have created humans in his image, yet God is also said to be omnipresent, and an omnipresent being would have no image. And God is said to guide individuals through difficult circumstances, yet God is also said to be all-powerful, and an all-powerful being would have brought about those circumstances in the first place.

One reason that notions of God are plagued with conceptual tensions is that God is attributed both anthropomorphic properties (e.g., “listens to prayers”) and non-anthropomorphic properties (e.g., “knows everything”). These tensions can be resolved only if some properties are privileged over others—if, for instance, the anthropomorphic properties are viewed as metaphors and the non-anthropomorphic properties as literal descriptions. Barrett and Keil investigated the extent to which American adults engage in such a practice by comparing their self-professed beliefs about God to the beliefs they unintentionally revealed in a story-recall task [8]. All participants claimed that God is omniscient and omnipresent when asked directly, but many drew anthropomorphic inferences on the story-recall task that contradicted such claims. For example, participants frequently mistook the statement “God was pleased by seeing the girl put the bird in its nest” for the statement “God was aware of the girl’s deed and was pleased by it” in the story-recall task even though the former, but not the latter, implies that God must perceive an event in order to be aware of it. Likewise, participants frequently mistook the statement “When the woman awoke, God had already left” for the statement “When she woke, she saw no one” even though the former, but not the latter, implies that God occupies a discrete location in space.

These findings suggest that anthropomorphic descriptions of God may be rejected at an explicit level but still influence how we reason about God at an implicit level, particularly in the context of stories. Still, the participants in Barrett and Keil’s study varied widely in how often they reasoned about God anthropomorphically. Participants’ accuracy at differentiating anthropomorphic descriptions of God from non-anthropomorphic ones ranged from 27% to 91%. Although Barrett and Keil acknowledged such differences, they did not explore them further. Individual differences in the anthropomorphization of God have been documented in several subsequent studies [9–13], but their origins and consequences remain unclear.

In the present study, we sought to understand variation in God concepts by assessing the beliefs and practices such concepts support. We hypothesized that, because public representations of God are ambiguous and inconsistent (as a whole), individual differences in God concepts may reflect differences in how people make sense of these representations. And because representations of God are embedded in a larger discourse about God—discourse on what God does, where God resides, who God interacts with, how God should be worshipped, and so forth—individual differences in God concepts may reflect individual differences in the interpretation of a wide range of religious ideas.

One reason to suspect that individuals who hold different God concepts also hold different God-related beliefs is that correlations between concepts and beliefs have been documented in several other domains of knowledge [14–15]. In physics, different concepts of force are correlated with different beliefs about acceleration, momentum, and inertia [16]. In chemistry, different concepts of matter are correlated with different beliefs about mass, weight, and density [17]. And in biology, different concepts of evolution are correlated with different beliefs about adaptation, speciation, and extinction [18].

These correlations arise because different people hold different conceptions of the same phenomena [19]. In physics, some people view forces as interactions between objects that change their speed and direction, whereas others view them as an internalized push or pull that keep an object in motion. In chemistry, some people view matter as collections of microscopic particles, whereas others view matter as discrete, homogenous units. And in biology, some people view evolution as the selective propagation of within-species variation, whereas others view evolution as the cross-generational transformation of a species’ underlying essence. Across domains, the correct view and the incorrect view are mutually incompatible, but each view is internally consistent and inferentially potent. Our understanding of natural kinds is organized in coherent networks of causal-explanatory beliefs [20–22], regardless of whether that understanding accurately represents reality. It is an open question whether our understanding of “supernatural kinds,” such as God, is organized in the same manner.

Here, we investigate whether people who view God as a human-like being reason about theological matters in a qualitatively different way from those who view God as an abstract entity. No research on God concepts has yet addressed this question because no research has looked for correspondences between people’s God concepts and their personal theologies. God concepts have been studied in relation to cognitive dispositions [23], moral dispositions [24], social attitudes [25], aggression [26], and wellbeing [27] but not in relation to the theological beliefs they support. Some research has sought to characterize variation in God concepts across ages or populations [28–33], but that research has focused on differences between individuals rather than correlations within an individual. Such correlations would imply not only that resolving the ambiguity inherent in God’s public representations has different consequences for different people but also that religious beliefs are organized in a theory-like manner.

To sketch the landscape of beliefs and practices surrounding anthropomorphic God concepts, we administered structured interviews on several religious topics: God’s properties and activities, other supernatural beings associated with God (angels and Satan), supernatural places associated with God (Heaven and Hell), God’s role in the origin of the universe, God’s role in the origin of humans, God’s relation to human suffering, and God’s relation to human misdeeds. We also surveyed our participants on their experiences with prayer and worship, and the factors that influenced their religious development.

We analyzed participants’ responses for evidence that they hold a concrete construal of the topic at hand, with the expectation that anthropomorphic God concepts engender more concrete beliefs. This connection can be demonstrated only if participants vary both in their God concepts and in their religious beliefs. Accordingly, we surveyed participants from a variety of religious backgrounds and with varying levels of religious conviction. We explore one important difference among our participants—whether they believe in God or not—but we do not delve into differences among other subpopulations. Rather, our focus is on whether variation in God concepts and God-related beliefs cohere. Coherence was assessed by correlating participants’ propensity to anthropomorphize God, as indexed by a property-attribution task, with the concreteness of their religious beliefs. Coherence was also assessed by correlating participants’ beliefs across different religious topics and by exploring the underlying structure of those beliefs with a Principle Components Analysis.

Several patterns of coherence are possible. At one extreme, God concepts and God-related beliefs may not cohere at all. This possibility is arguably the most likely given that people acquire their religious beliefs from a variety of sources in a variety of contexts at a variety of times, creating many opportunities for inconsistency and contradiction. At the other extreme, God concepts and God-related beliefs may cohere perfectly, such that individuals who hold anthropomorphic concepts interpret every religious topic differently from those who hold abstract ones. Strong patterns of coherence have been observed in scientific domains, when comparing novices to experts [16,34], but such patterns weaken as novices gain relevant knowledge [35–36]. A third possibility is that God concepts will cohere with God-related beliefs but along more than one dimension, indicating that anthropomorphic God concepts support distinct clusters of beliefs. To preview our results, we find evidence of the last pattern. God concepts correlate with all beliefs surveyed, but they relate to some beliefs (e.g., beliefs about angels and Satan) differently than others (e.g., beliefs about cosmogenesis and anthropogenesis).

## Method

This study was reviewed by the Occidental College IRB and approved as Shtu-D8087.

### Participants

The participants were 275 undergraduates at Occidental College recruited from introductory psychology courses and compensated with extra credit in those courses. Participants were directed to an online questionnaire that took approximately 45 minutes to complete. Participants reported a wide range of religious affiliations: 26% Protestant, 19% Catholic, 11% Jewish, 3% Buddhist, 2%, Hindu, 2% Unitarian, 1% Muslim, 1% something else (Wiccan, Taoist, Navajo), and 36% unaffiliated.

Fifty-eight percent of participants (*n* = 160) claimed that God exists, and 42% claimed that God does not exist (*n* = 115). The former are referred to as “theists,” and the latter “atheists.” We report mean differences between theists and atheists on key measures, but we include all participants in analyses of the relation between God concepts and God-related beliefs and practices. Atheists provided responses to all questions, just as theists did, and their responses proved equally codable. Atheists may not have endorsed religious ideas, but they provided interpretations of those ideas nonetheless. For simplicity’s sake, we refer to the responses provided by both theists and atheists as “beliefs,” but we mean belief in the sense of mental proposition rather than personal conviction.

### Procedure

Each participant completed a 108-item questionnaire intended to cover the breadth and depth of participants’ personal theologies. These items are discussed in conjunction with participants’ responses in the Results section. A handful of items yielded ambiguous or unvarying responses and were dropped from the final analysis for brevity’s sake. They include three questions about the origin of supernatural beings (e.g., “Where did God come from?”, to which most participants responded “I don’t know” or “God has always existed”) and 16 questions about the origin and function of the soul.

Participants began the survey by reporting whether they believe in the existence of God, angels, Satan, Heaven, and Hell and rating how confident they were on a seven-point scale. Participants then decided whether three of those entities—God, angels, and Satan—could be attributed each of twelve human properties. Four of the properties were psychological (*dreams*, *sees*, *talks*, *thinks*), four were biological (*eats*, *grows*, *sleeps*, *sneezes*), and four were physical (*gets cold*, *gets wet*, *sits*, *stretches*). These properties were selected from previous research (Shtulman, 2008; Shtulman & Lindeman, 2016), which validated that all twelve are seen as characteristic of humans. The properties were arranged in alphabetical order and were presented as yes-or-no questions, as in “Does God sleep?” or “Do angels get cold?”.

### Coding

Several survey questions elicited verbal responses, which were coded for evidence that participants interpreted the topic in concrete terms. For instance, questions about what a supernatural being looks like were coded for evidence that participants do, in fact, think the being has a physical appearance, whatever that appearance might be. Our coding hinged on whether participants expressed concrete ideas, regardless of the details. A participant who described angels as “white with wings” and a participant who described them as “beautiful, tall super humans” were both coded as providing concrete responses, even though the details were different. Likewise, a participant who described angels as “beyond human perception” and a participant who described them as “pure energy” were both coded as *not* providing concrete responses. In this manner, our analyses constitute a course-grained, first-pass attempt at differentiating concrete theologies from abstract ones. Additional, detail-specific coding is planned for the future.

The coding criteria for each question are described in conjunction with the question. All responses were coded by two independent judges blinded to the identity of the participants. One coder was also blinded to the study’s hypotheses. Overall agreement between judges was 92%, and disagreements were resolved through discussion.

## Results

### Measures of conceptualization

Our primary question was whether anthropomorphic God concepts correlate with concrete interpretations of religious ideas. Thus, we begin by describing our measures of anthropomorphism, followed by our measures of concreteness. The latter were analyzed by topic, with the topics being (1) God, (2) angels, (3) Satan, (4) Heaven, (5) Hell, (6) cosmogenesis and anthropogenesis, (7) suffering and misdeeds, and (8) prayer and worship. We summed the number of concrete responses provided for each topic and then compared those composites to our measure of anthropomorphism, controlling for participants’ confidence in God’s existence. We also compared our composites directly, to assess whether concrete interpretations of religious ideas hang together on their own, apart from their relation to God concepts.

#### Anthropomorphism

**Fig 1:**
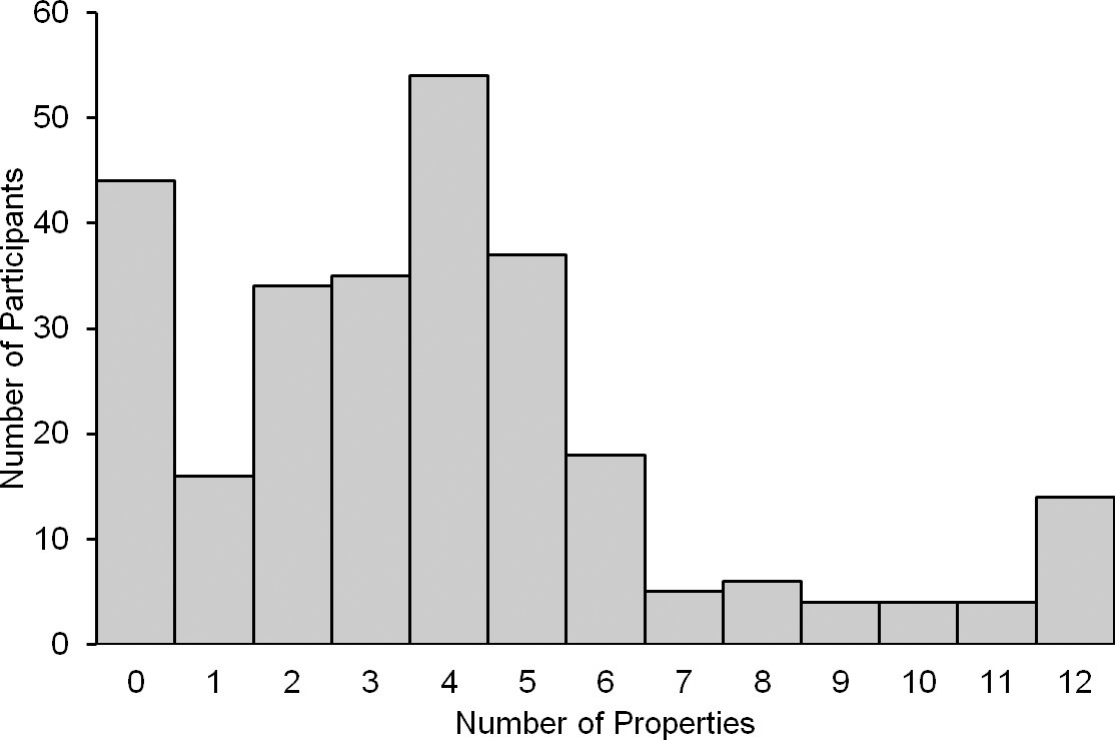
Number of participants who attributed 0–12 human properties to God.

At the beginning of the survey, participants decided whether God can be attributed each of twelve human properties. Participants’ attributions ranged from 0 to 12, with a mean attribution of 3.9 and a standard deviation of 3.1 (see Fig 1). Theists attributed more properties to God than atheists (*M* = 4.6 vs. *M* = 2.8, *t*(273) = 5.01, *p* < .001), but most atheists (51%) attributed at least three properties and some (12%) attributed six or more.

Property attributions varied by domain, with participants attributing an average of 2.4 psychological properties (out of four) but only 0.6 biological properties and 0.9 physical properties. Psychological attributions reliably outnumbered both biological attributions (*M* = 2.4 vs. *M* = 0.6, *t*(273) = 22.78, *p* < .001) and physical attributions (*M* = 2.4 vs. *M* = 0.9, *t*(273) = 22.48, *p* < .001), which replicates the previous finding that God is viewed primarily as an intentional agent [37,12]. That said, participants’ attributions were highly correlated across domains. Psychological attributions correlated with biological attributions (*r* = .43, *p* < .001); biological attributions correlated with physical attributions (*r* = .82, *p* < .001), and physical attributions correlated with psychological attributions (*r* = .62, *p* < .001). When participants anthropomorphized God, they tended to do so across all domains, despite baseline differences between psychological attributions and other attributions.

#### Beliefs about God

The first part of the questionnaire probed participants’ beliefs about God’s appearance and activities. Participants answered three open-ended questions: “What is God?”, “What does God look like?”, and “What does God do?”. Responses were analyzed collectively for evidence that participants believe (1) God watches over or rules over the world, (2) God intervenes in human affairs, and (3) God has a physical appearance. Responses indicative of belief 1 include “He controls everything that occurs on earth,” “[God] watches over the world, sees that things follow his plan.” Responses indicative of belief 2 include “God watches over his believers and interacts with them on a personal level,” “God reins over all living things. He loves, gets angry, judges, etc.” And responses indicative of belief 3 include “[God looks] like us, like ordinary human beings,” “if we are made in the image of God, then we must look something like God.”

Overall, 59% of participants stated that God watches over or rules over the world, 56% that God intervenes in human affairs, and 54% that God has a physical appearance. Participants were also asked whether God answers prayers; 42% said yes and 58% said no. In sum, participants provided an average of 2.1 concrete beliefs about God out of four (*SD* = 1.3). Differences in the number of concrete responses provided by theists and atheists can be found in the Supplemental Materials, along with correlations between providing each response and anthropomorphizing God. The same analyses for other topics are included in the Supplemental Materials as well.

#### Beliefs about angels

Following the questions about God, participants answered essentially the same questions about angels, including twelve property-attribution questions and three open-ended questions: “What are angels?”, “What do angels look like?”, and “What do angels do?”. Responses to the open-ended questions were coded collectively for evidence that participants believe (1) angels are God’s servants or helpers, (2) angels intervene in human affairs, and (3) angels have a physical appearance. Responses indicative of belief 1 include “they serve God,” “those who assist God in his wishes,” “they do God’s bidding.” Responses indicative of belief 2 include “[angels] intercede and interact with man,” “[angels] intervene in people’s lives, protect them.” And responses indicative of belief 3 include “angels look like people dressed in white with wings and halos,” “they are beautiful/handsome with pale skin, red cheeks, and flowing blonde hair.”

Half of participants claimed that angels exists, and half claimed they do not. Forty-nine percent of participants stated that angels are God’s helpers or servants; 66% stated that angels intervene in human affairs; and 81% stated that angels have a physical appearance. These four responses were summed, yielding an average composite of 2.5 concrete beliefs (*SD* = 0.9). With respect to property attributions, participants’ attributions to angels were highly correlated with their attributions to God (*r* = .55, *p* < .001). These data were not, however, included in the composite scores, as the variance in twelve property attributions would have swamped the variance in the other four responses. More details on property attributions to angels can be found in the Supplemental Materials.

#### Beliefs about Satan

The questions asked about angels were repeated for Satan and coded for evidence that participants believe (1) Satan is God’s enemy, (2) Satan intervenes in human affairs; and (3) Satan has a physical appearance. Responses indicative of belief 1 include “Satan is God’s greatest enemy,” “[Satan is] the evil being that opposes God.” Responses indicative of belief 2 include “[Satan] tempts people to do immoral things,” “[Satan] deceives, tricks, and does everything possible to have people turn away from God.” And responses indicative of belief 3 include “He looks like a human being that is all red and has a tail,” “Satan is a dark, ominous figure, always surrounded by flames.”

Thirty-percent of participants claimed that Satan exists, and 70% claimed that he does not. Thirty-two percent of participants stated that Satan is God’s enemy; 57% stated that Satan intervenes in human affairs; and 72% stated that Satan has a physical appearance. In sum, participants provided an average of 1.9 concrete responses about Satan out of a possible four (*SD* = 1.1). As with angels, property attributions to Satan were strongly correlated with property attributions to God (*r* = .65, *p* < .001), but property attributions were excluded from the four-item composite. Additional analyses can be found in the Supplemental Materials.

#### Beliefs about Heaven and Hell

Participants beliefs about Heaven were elicited with the questions “What is Heaven?”, “Where is Heaven?”, “What does Heaven look like?”, and “What is done in Heaven?” Responses were coded collectively for evidence that participants believe (1) Heaven has a physical location, (2) Heaven has a physical appearance, and (3) human activities continue in Heaven. The same questions were asked about Hell, and the same coding scheme was applied.

Responses indicative of belief 1 include “Heaven is in the sky,” “Heaven is somewhere above us in the clouds,” “Hell is underground,” “Hell is in the center of the earth.” Responses indicative of belief 2 include “Heaven has a lot of clouds and light,” “Heaven is white and spacious,” “Hell is dark, dirty, and filled with fire,” “[Hell is] a rocky cave with lots of fire and bad lighting.” And responses indicative of belief 3 include “people in Heaven see their family who have passed away and look over those who still live,” “People [in Heaven] live as they do among Earth, yet they are happier and friendly,” “[in Hell] you are forced to labor for the rest of eternity,” “[in Hell] people are punished and tortured and live terrible lives.”

Forty-nine percent of participants claimed that Heaven exists, and 51% claimed that it does not; 33% claimed that Hell exists, and 67% claimed that it does not. As for Heaven and Hell’s characteristics, 59% stated that Heaven has a physical location, and 61% stated that Hell does; 73% stated that Heaven has a physical appearance, and 77% stated that Hell does; 71% stated that Heaven is characterized by human activities, and 78% stated that Hell is. Across these four items, participants provided an average of 2.5 concrete responses about Heaven (*SD* = 1.2) and 2.5 concrete responses about Hell (*SD* = 1.1).

#### Beliefs about cosmogenesis and anthropogenesis

The next part of the survey probed participants’ beliefs about God’s role in the origin of the universe (cosmogenesis) and the origin of human beings (anthropogenesis). Participants’ beliefs about cosmogenesis were elicited with the questions “Do you believe that God created the universe?”, “Do you believe that the universe was created in the Big Bang?”, and “If you answered ‘yes’ to both questions, how do you resolve the apparent inconsistency between these two ideas?” Participants’ beliefs about anthropogenesis were elicited with the questions “Do you believe that God created human beings?”, “Do you believe that human beings evolved from other organisms?”, and “If you answered ‘yes’ to both questions, how do you resolve the apparent inconsistency between these two ideas?”

With respect to cosmogenesis, 13% of participants claimed that the universe was created by God alone, 49% by the Big Bang alone, and 38% by both God and the Big Bang. Among those who endorsed both God and the Big Bang, 67% justified their claim by appealing to a dual process (e.g., “God created the Big Bang,” “God initiated the Big Bang and then perfected and sculpted the world,” “creating the universe from a singularity is still creating the universe”). With respect to anthropogenesis, 10% of participants claimed that human beings were created by God alone, 60% by evolution alone, and 30% by both God and evolution. Among those who endorsed both God and evolution, 78% appealed to a dual process (e.g., “God created the infrastructure of life within which evolution occurs,” “evolution is the means by which God created humans”). While scientists may not endorse dual-process views, their frequency is consistent with previous research demonstrating that people often explain natural phenomena by appealing to both scientific and supernatural processes [38].

Participants who claimed that God played a role in cosmogenesis or anthropogenesis, either direct or indirect, were coded as holding a concrete interpretation of God as a creator. Across the two forms of genesis, participants provided an average of 0.9 concrete responses (*SD* = 0.9).

#### Beliefs about suffering and misdeeds

In the next section, participants answered questions about God’s relation to human suffering and human misdeeds. These beliefs were elicited by asking participants to reason about two theological problems, traditionally known as the “problem of evil” and the “problem of omniscience.” The problem of evil was raised with the questions “Do you believe that God is omnipotent (all-powerful)?”, “Do you believe that God is omnibenevolent (all-good)?”, and “If you answered ‘yes’ to both questions, why do you think God allows (or fails to prevent) human suffering?”. The problem of omniscience was raised with the questions “Do you believe that God is omniscient (all-knowing)?”, “Do you believe that God holds human beings responsible for their actions?”, and “If you answered ‘yes’ to both questions, why do you think God holds human beings responsible for actions God knows they will make?”.

With respect to the problem of evil, 51% of participants denied that God is omnipotent, and 45% denied that God is omnibenevolent. The remaining participants (39%) affirmed that God is both omnipotent and omnibenevolent and were thus prompted to explain human suffering. Of those, 63% claimed that God uses suffering to teach or punish (e.g., “overcoming suffering can be a challenge that can bring about learning, change, and growth,” “God does not want humans to suffer but allows suffering to teach humans the value of free will and decision making”) and 36% claimed that God allows suffering as part of a larger plan (e.g., “He has an ultimate plan for His creations that we, as humans, cannot possibly understand,” “God has a plan laid out for everything and everyone. … Bad things happen so that good things can come about from them”).

With respect to the problem of omniscience, 41% of participants denied that God is omniscient, and 43% denied that God holds humans responsible for their actions. The remaining participants (45%) claimed that God is both omniscient and an arbiter of human actions. In explaining why God holds humans responsible for actions he knows they will make, 58% claimed that God gave humans free will and thus the capacity to behave immorally (e.g., “The gift of free will to make our own choices, to choose to love other people and Himself is a wonderful manifestation of God’s love for humans,” “God knows what decisions we will make but He granted us free will”), and 24% claimed that God wants humans to learn from their mistakes (e.g., “God just wants us to learn from our mistakes and see if we’re loyal to him the next time,” “God only places us on this Earth so that we can learn for ourselves how to survive and live with each other; He expects that there will be some people who will make mistakes”).

In sum, some participants viewed human suffering and human misdeeds as secular phenomena, outside of God’s control or purview, while others viewed suffering as divinely ordained and misdeeds as divinely punishable. The latter views are reminiscent of the ideology previously characterized as “belief in a just world” [39], with God seen as the reason the world is just [40]. Participants who reported that God causes or allows suffering were coded as holding a concrete interpretation of God’s role in human suffering, and participants who reported that God holds humans responsible for their misdeeds were coded as holding a concrete interpretation of God’s role in human misdeeds. In total, participants provided an average of 0.8 concrete beliefs with respect to these issues (*SD* = 0.8).

#### Religious practices and development

The final part of the survey included five questions about participants’ religious practices: “How often do you pray?”, “What do you do when you pray?”, “What do you typically pray for or about?”, “What do you do (if anything) to increase the likelihood that God will answer your prayers?” and “How often do you attend religious services?”. Forty-eight percent of participants reported that they pray at least occasionally, and 15% reported that they pray once or more per day. Of those who pray, 27% reported that they engage in particular behaviors (e.g., “close my eyes and say Amen,” “fold my hands and get on my knees,” “make the sign of the cross”), and 54% reported that they petition God for specific, tangible outcomes (e.g., “good grades in my classes,” “a job position,” “health, strength, and success”). Finally, 67% of participants reported that they attend religious services at least occasionally, and 15% reported that they attend religious services once a week or more.

Four of these responses were coded as evidence of concrete religious practices: praying daily, praying in a ritualized manner, praying for specific outcomes, and attending religious services weekly. Of these, participants reported engaging in an average of 0.7 (*SD* = 1.0).

Also included at the end of the survey were three questions about participants’ religious development: “From whom did you acquire most of your religious beliefs?”, “What kind of formal religious instruction have you had, if any?”, and “How have your religious beliefs changed over time, if at all?”. Sixteen percent of participants claimed to have acquired their beliefs from a religious authority, such as a priest, rabbi, or religious institution; 80% claimed to have acquired their beliefs from family and friends; and 24% claimed to have acquired their beliefs on their own, through reading or reflection. Sixty-five percent reported having had some kind of religious instruction, and 79% claimed their beliefs had changed over time. Of those whose beliefs had changed, 18% claimed that their beliefs had grown stronger or more elaborate (e.g., “I believe in God more,” “my faith has strengthened significantly”), and 56% claimed their beliefs had grown weaker or less dogmatic (“I have lost faith in the power of organized religion,” “I am less concerned with literal interpretations of religious texts”).

Anthropomorphizing God was associated with acquiring beliefs from a religious authority (*r* = .12, *p* < .05) and developing stronger beliefs over time (*r* = .21, *p* < .001). Developing weaker beliefs was also correlated with anthropomorphism but in the opposite direction (*r* = -.19, *p* < .01). This set of correlations runs counter to the notion of “theological correctness” [41]. Anthropomorphic God concepts are not theologically correct, but those who hold them report having had more religious instruction. They also report that their beliefs have grown stronger with time, despite overt contradictions between the properties of humans (e.g., limited knowledge, limited power, limited vitality) and the properties of God (e.g., omniscience, omnipotence, immortality). More information about participants’ religious development and religious affiliations can be found in the Supplemental Materials.

#### Composite scores

Means for the eight composites are displayed in Table 1 as a function of whether participants identified as theists or atheists. Theists’ means were higher than atheists’ means for seven of eight topics: God (*M* = 2.4 vs. *M* = 1.7, *t*(273) = 4.72, *p* < .001), angels (*M* = 2.7 vs. *M* = 2.1, *t*(273) = 5.18, *p* < .001), Satan (*M* = 2.1 vs. *M* = 1.6, *t*(273) = 3.83, *p* < .001), Heaven (*M* = 2.8 vs. *M* = 2.2, *t*(273) = 4.46, *p* < .001), cosmogenesis and anthropogenesis (*M* = 1.4 vs. *M* = 0.2, *t*(273) = 14.24, *p* < .001), suffering and misdeeds (*M* = 1.1 vs. *M* = 0.3, *t*(273) = 9.81, *p* < .001), and religious practices (*M* = 1.1 vs. *M* = 9.84, *t*(273) = 0.1, *p* < .001). The only topic for which theists and atheists revealed a similar number of concrete responses was Hell. Overall, theists provided an average of 16.2 concrete responses, and atheists provided an average of 10.6 (*t*(273) = 10.01, *p* < .001). While this difference is reliable, it constitutes fewer than six responses (out of 28), indicating that many atheists reason about religious matters as concretely as theists do.

**Table 1:** Mean number of concrete responses provided by theists and atheists, as well as their correlations with anthropomorphic God concepts controlling for confidence in God’s existence.

Topic	Range	Theists	Atheists	Difference	Partial *r*
God	0–4	2.4	1.7	0.7***	.36***
Angels	0–4	2.7	2.1	0.6***	.31***
Satan	0–4	2.1	1.6	0.5***	.16**
Heaven	0–4	2.8	2.2	0.6***	.33***
Hell	0–4	2.6	2.4	0.2	.26***
Cosmogenesis and anthropogenesis	0–2	1.4	0.2	1.2***	.27***
Suffering and misdeeds	0–2	1.1	0.3	0.9***	.25***
Prayer and worship	0–4	1.1	0.1	1.0***	.23***

*p < .05.

**p < .01.

***p < .001.

### Measures of coherence

#### Correlations between anthropomorphism and composite scores

Participants’ composite scores consistently correlated with their propensity to anthropomorphize God, as shown in Table 1. These correlations control for participants’ confidence in God’s existence, as more confident individuals may have provided more informative responses in general. Even with this control, the correlations remained significant, averaging 0.27 in magnitude. The more participants anthropomorphized God, the more concretely they reasoned about several distinct religious topics.

#### Correlations among composite scores

The eight composites were entered into a correlation matrix, along with our measure of anthropomorphism (total number of properties attributed to God). The resulting correlations are displayed in Table 2, above the matrix’s diagonal. All 36 correlations were positive, and 33 of the 36 were significant, with an average effect size of *r* = .32. This pattern indicates that participants who provided concrete responses for one topic tended to provide concrete responses for all other topics. One concern with this analysis is that it may have been driven by global differences between theists and atheists rather than topic-specific differences in religious beliefs. To address this concern, we repeated the analysis with only the theists’ scores. Those correlations are presented below the diagonal in Table 2. Thirty-five of the 36 correlation remained positive, and 28 remained significant, with an average effect size of *r* = .26. Theists’ responses thus proved coherent even when analyzed by themselves.

**Table 2:** Correlations between anthropomorphization of God (1) and concrete beliefs and practices about other religious matters (2–9). Correlations above the diagonal are for the entire sample; correlations below the diagonal are for theists only.

Measure	1	2	3	4	5	6	7	8	9
1. Total property attributions	—	.39***	.34***	.19**	.34***	.27***	.31***	.29***	.28***
2. God	.30***	—	.42***	.35***	.40***	.31***	.34***	.46***	.29***
3. Angels	.34***	.38***	—	.39***	.41***	.36***	.28***	.30***	.25***
4. Satan	.12	.37***	.33***	—	.31***	.52***	.26***	.33***	.25***
5. Heaven	.24**	.31***	.38***	.29***	—	.53***	.18**	.22***	.10
6. Hell	.20*	.23**	.25**	.58***	.44***	—	.08	.13*	.11
7. Cosmogenesis and anthropogenesis	.22**	.29***	.14	.23**	.04	.10	—	.59***	.54***
8. Suffering and misdeeds	.25**	.47***	.23**	.33***	.10	.11	.50***	—	.47***
9. Prayer and worship	.24**	.27***	.16*	.20*	-.04	.13	.34***	.33***	—

*p < .05.

**p < .01.

***p < .001.

#### Principal components analysis

To further explore the coherence among responses, we submitted our eight composites to a Principal Component Analysis with Varimax rotation. This analysis revealed two components with Eigenvalues greater than 1 (i.e., two components capable of explaining more variance than explained by any individual variable). The first component explained 29.7% of the variance in participants’ responses, and the second explained 27.1%, for a total of 56.8%.

Six variables loaded highly (>.3) on the first component: properties attributions to God (.45), beliefs about God (.55), beliefs about angels (.64), beliefs about Satan (.64), beliefs about Heaven (.78), and beliefs about Hell (.83). Five variables loaded highly on the second component: property attributions to God (.39), beliefs about God (.46), beliefs about anthropogenesis and cosmogenesis (.84), beliefs about suffering and misdeeds (.79), and religious practices (.78). It would appear that the first component represents how concretely participants conceptualize religious entities and religious places, whereas the second represents how concretely participants reason about God’s role in worldly affairs, including participants’ own lives (given the association with religious practices).

This analysis was repeated for theists alone and revealed the same results. Two components emerged capable of explaining the majority of variance in participants’ responses (26.5% for the first component and 25.0% for the second), and the loadings for each component mirrored the original loadings. Six variables loaded highly on the first component (properties attributions to God, .35; beliefs about God, .46; beliefs about angels, .61; beliefs about Satan, .67; beliefs about Heaven, .78; and beliefs about Hell, .78), and five loaded highly on the second (property attributions to God, .40; beliefs about God, .56; beliefs about anthropogenesis and cosmogenesis, .76; beliefs about suffering and misdeeds, .78; and religious practices, .68). A two-component solution appears to capture the variance in participants’ concrete responses even when the lower end of that variance (from atheists) is removed.

#### Correlations between anthropomorphism and total scores

**Fig 2:**
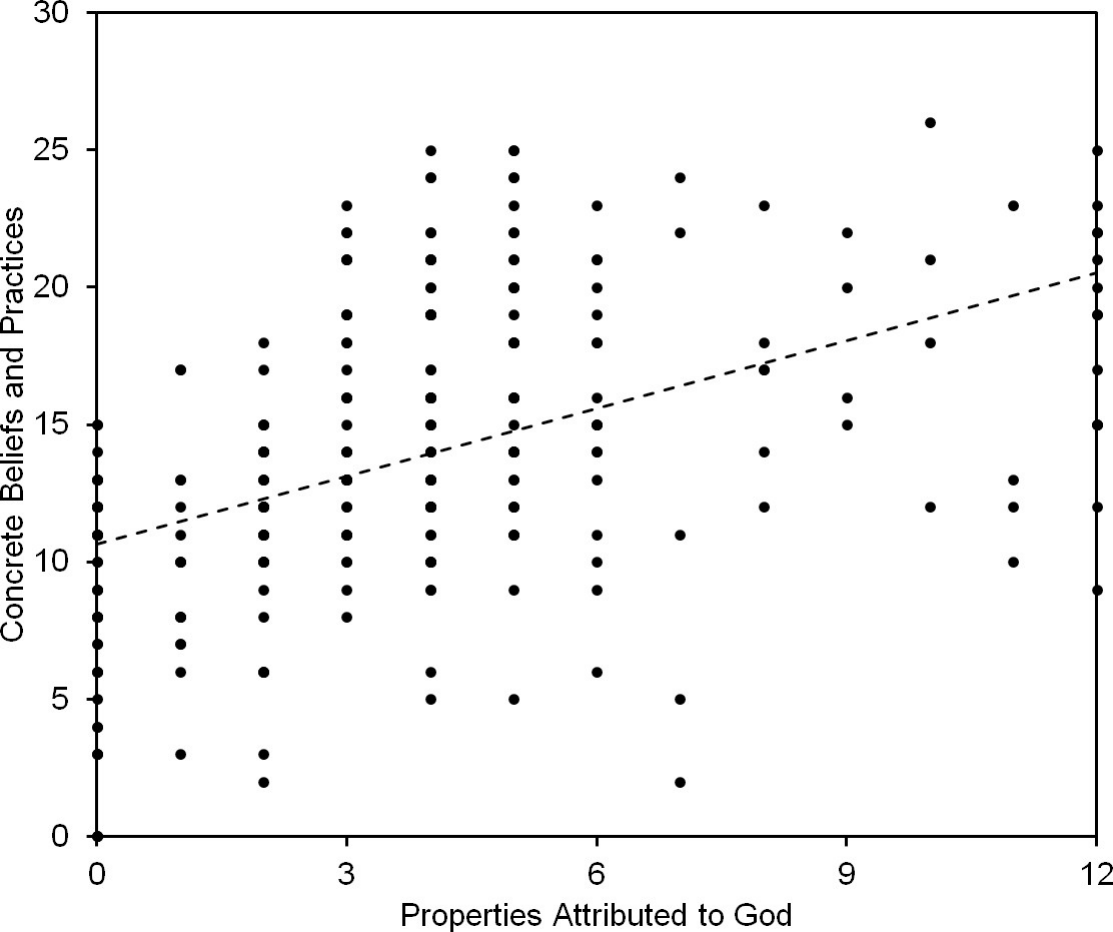
Relation between human properties attributed to God and concrete beliefs and practices endorsed.

In one final analysis, we summed the number of concrete responses provided across the entire survey (ranging from 0 to 26) and compared those sums to the number of properties attributed to God (ranging from 0 to 12). This analysis is displayed in Fig 2. The two measures were strongly correlated (*r* = .47, *p* < .001), and the scatterplot between them reveals no indication of a discontinuity. Participants’ interpretations of religious ideas fall along a continuum from concrete to abstract, corresponding to how strongly those participants anthropomorphize God.

## Discussion

Within Western culture, public representations of God range from highly anthropomorphic to highly abstract, allowing for different interpretations of the same being. Here, we explored the relationship between participants’ endorsement of an anthropomorphic conception of God and their associated beliefs and practices. Overall, it was found that participants’ propensity to anthropomorphize God was correlated with their propensity to (a) view God as a palpable influence on human affairs, (b) anthropomorphize angels and Satan; (c) spatialize Heaven and Hell, (d) assume that God played a role in the origin of the universe and the origin of humans, (e) assume that God allows human suffering and punishes human misdeeds, and (f) engage in traditional religious activities, like prayer and worship.

Underlying these correlations were two dimensions of conceptualization: a dimension relating to how concretely participants conceive of supernatural entities and a dimension relating to how concretely participants conceive of God’s role in the natural world. The first dimension encompasses the beliefs that angels, Satan, Heaven, and Hell actually exist, that angels and Satan are physical beings that interact with God, and that Heaven and Hell are physical locations occupied by these beings. The second dimension encompasses the beliefs that God created the universe and the humans within, that God allows human suffering and punishes human misdeeds, and that one can communicate with God through prayer and worship.

Both sets of beliefs make sense only if God possesses human properties because human properties are required for activities like collaboration (with angels), conflict (with Satan), habitation (of Heaven), intentional design (of humans and the universe), teaching (through the infliction of suffering), punishment (of misdeeds), and communication (with believers). Anthropomorphic conceptions of God bring with them an entire ontology for interpreting religious ideas—the ontology of human beings and human affairs [42]. This ontology appears to guide reasoning about supernatural phenomena (dimension 1), as well as reasoning about the supernatural underpinnings of natural phenomena (dimension 2). That said, the two dimensions proved separable. Some participants conceived of supernatural entities more concretely than they conceived of God’s role in the natural world, and others did the opposite. It is an open question how often the two dimensions diverge and whether one dimension is more basic than the either, either conceptually or developmentally. Previous research on children’s God concepts indicates that children hold more anthropomorphic concepts than adults [43,10–11], but it is unknown whether children’s religious beliefs in general are also more concrete.

It is also unknown whether individuals who do *not* anthropomorphize God conceive of God and God-related matters using some other ontology. In the present study, abstract responses were identified by the absence of concrete details rather than the presence of an alternative criterion, which limits our ability to interpret the abstract end of the abstract-to-concrete continuum displayed in Fig 2. Further analysis is needed to determine whether abstract theologies can be given a positive characterization, such as an energy-based theology, a force-based theology, or an intention-based theology (predicated on the idea that God is an intentional agent but not a physical being). While notions of human existence and human affairs provide readily-accessible models for concrete interpretations of religious ideas, it is unclear what models are available for interpreting the same ideas abstractly.

Atheists, on average, expressed more abstract (or less concrete) views than theists, which might be expected if atheists have not considered religious ideas in much depth. But the atheists in our study appear to have considered religious ideas and then rejected them. Seventy-four percent claimed that their religious beliefs had changed over time, with 54% claiming that their beliefs had weakened over time. Given the prevalence of religion in society at large, most adult atheists were likely raised in religious households and, accordingly, developed interpretations of religious ideas during their upbringing. Those interpretations were less concrete than theists’, but they were not entirely abstract. Across the 28 responses that fed into our composite variables, atheists provided only 5.6 fewer than theists—a mere 20% difference.

The interaction between how one conceives of religious ideas and whether one accepts those ideas as true merits further study. One might predict that atheists’ interpretations of religious ideas should be more concrete than theists, since concrete interpretations are vulnerable to the kinds of skepticism-inducing contradictions noted in the Introduction (e.g., why should we pray to God if God knows everything?), but the opposite was found: atheists interpreted religious ideas more abstractly than theists. One reason for this difference may be that the atheists in our study who were once theists abandoned concrete interpretations earlier in their religious development, as an attempt to maintain belief, but ultimately found abstract interpretations untenable as well. Longitudinal studies of religious development are needed to assess the role of conceptualization in maintaining—or losing—belief.

### Limitations and future directions

A primary limitation of the current study is that we relied on self-report. Self-report is a well-worn means of measuring God concepts [44–45], but the beliefs revealed through self-report may differ from those revealed by more subtle measures, such as story recall [8] or speeded sentence verification [46–47]. The latter measure entails asking participants to verify statements about God as quickly as possible. Some statements are consistent with core intuitions about humans (e.g., “God can hear what I say out loud”), and others are inconsistent with those intuitions (e.g., “God can hear what I say to myself”). Participants verify the latter type of statement more slowly and less accurately than they verify the former, implying that core intuitions about humans actively conflict with explicit beliefs about God.

It is an open question how explicit measures of anthropomorphization, like those used in the present study, relate to more implicit measures. One possibility is that the two measures stand in opposition, such that the less participants anthropomorphize God in explicit tasks, the more cognitive conflict they demonstrate in implicit tasks. Another possibility, however, is that the two measures tap different psychological constructs, with explicit measures tapping personally-endorsed beliefs about God and implicit measures tapping everything one knows about God, endorsed or not. On this view, *all* participants would experience conflict in an implicit task because all participants are aware that God has both abstract and anthropomorphic properties. Research in this vein can help establish the role of collateral religious beliefs—i.e., beliefs about angels, Satan, Heaven, Hell, and so forth—in determining which God concepts are prioritized and under what circumstances.

Another limitation of our study is that our methods are correlational. We have argued that anthropomorphic God concepts breed concrete theologies (in accordance with the theory-theory view of conceptual development), but the opposite might be true. For instance, learning that angels and Satan are essentially human in appearance and behavior may lead people to infer that God has human properties as well. Or perhaps all three beings are learned about together, as part of a concrete theology acquired whole-cloth from religious instruction rather than derived through the lens of an anthropomorphic conception of God.

While this interpretation cannot be ruled out, there are many reasons to doubt it. First, the input people receive about theological matters is probably no less vague than the input they receive about God, leaving ample room for interpretation. Second, participants in the present study came from a variety of religious backgrounds, implying that the coherence we observed was not driven by a particular type of religious education. While Protestants were slightly more likely to anthropomorphize God than participants from other religions (*r* = .14, *p* < .05), the mean number of properties attributed to God by Protestants, Catholics, and Jews differed by less than one (4.6, 4.2, and 4.0, respectively). Third, participants were unlikely to have pondered all topics of our survey prior to their participation, yet the responses they generated were internally consistent. Such consistency implies that participants’ responses were derived from an underlying theory rather than repeated verbatim from previous instruction [48].

That said, future research could explore the development of personal theologies more directly. For instance, one could investigate the theologies of young children and chart how these theologies change over time. Alternatively, one could compare the theologies of different members of the same cultural unit, like the same church or family, to determine which dimensions of personal theologies are most likely to vary and which are not. As noted earlier, our goal in the present study was to test for coherence among a wide range of beliefs across a wide range of participants, and we found that participants’ beliefs varied coherently. Variance is required to establish coherence, but the source of the variance is a topic of investigation in its own right. Some social groups may hold more concrete beliefs than others, and identifying those groups would shed additional light on the nature and origin of such beliefs.

Research in this vein would not only increase our understanding of religious cognition but would also increase our understanding of the interaction between cognition and culture more generally. Religious ideas are one of the few types of ideas conveyed solely through culture, and studying the diverse ways in which we interpret those ideas promises to shed light on the constraints and capabilities of cultural transmission more generally.

## Supporting information

S1 Table Responses to questions about God by theists and atheists, plus correlations between responses and anthropomorphization of God. (PDF)

S2 Table Responses to questions about angels by theists and atheists, plus correlations between responses and anthropomorphization of God. (PDF)

S3 Table Responses to questions about Satan by theists and atheists, plus correlations between responses and anthropomorphization of God. (PDF)

S4 Table Responses to questions about Heaven and Hell by theists and atheists, plus correlations between responses and anthropomorphization of God. (PDF)

S5 Table Responses to questions about cosmogenesis and anthropogenesis by theists and atheists, plus correlations between responses and anthropomorphization of God. (PDF)

S6 Table Responses to questions about suffering and misdeeds by theists and atheists, plus correlations between responses and anthropomorphization of God. (PDF)

S7 Table Responses to questions about religious practices, affiliation, and development by theists and atheists, plus correlations between responses and anthropomorphization of God. (PDF)

S8 Table Correlations among psychological, biological, and physical properties attributions to God, angels, and Satan. (PDF)
