# Supplementary material for: Theories of God: Explanatory coherence in religious cognition
Source: PLoS One. 2018 Dec 26;13(12):e0209758. doi: 10.1371/journal.pone.0209758 (PMC6306263; doi:10.1371/journal.pone.0209758)
Supplement: S1 Table — (PDF) [file pone.0209758.s001.pdf]

S1 Table. Responses to questions about God by theists and atheists, plus correlations between responses and anthropomorphization of God.

	Mean			
Response	Theists	Atheists	Difference	Correlation
Beliefs				
God exists.	1.00	.00	NA	.29***
God rules/watches over the world.	.59	.58	.01	.21***
God intervenes in human affairs.	.59	.51	.07	.20***
God has a physical appearance.	.55	.52	.03	.31***
God answers prayers.	.67	.07	.60***	.29***
Attributions				
Psychological properties	2.8	1.9	0.8***	.82***
Biological properties	0.7	0.3	0.4**	.84***
Physical properties	1.1	0.6	0.6***	.93***
All properties	4.6	2.8	1.8***	NA

*p < .05, **p < .01, ***p < .001
