# Supplementary material for: Theories of God: Explanatory coherence in religious cognition
Source: PLoS One. 2018 Dec 26;13(12):e0209758. doi: 10.1371/journal.pone.0209758 (PMC6306263; doi:10.1371/journal.pone.0209758)
Supplement: S2 Table — (PDF) [file pone.0209758.s002.pdf]

S2 Table. Responses to questions about angels by theists and atheists, plus correlations between responses and anthropomorphization of God.

	Mean			
Response	Theists	Atheists	Difference	Correlation
Beliefs				
Angels exist.	.75	.15	.60***	.34***
Angels are God's servants/helpers.	.50	.48	.02	.17**
Angels intervene in human affairs.	.64	.69	-.05	-.01
Angels have a physical appearance.	.80	.82	-.02	.16*
Attributions				
Psychological properties	3.0	2.6	0.4*	.48***
Biological properties	1.0	0.9	0.1	.47***
Physical properties	1.6	1.3	0.3	.49***
All properties	5.6	4.8	0.8	.54***

*$p < .05$, **$p < .01$, ***$p < .001$
