# Supplementary material for: Theories of God: Explanatory coherence in religious cognition
Source: PLoS One. 2018 Dec 26;13(12):e0209758. doi: 10.1371/journal.pone.0209758 (PMC6306263; doi:10.1371/journal.pone.0209758)
Supplement: S3 Table — (PDF) [file pone.0209758.s003.pdf]

S3 Table. Responses to questions about Satan by theists and atheists, plus correlations between responses and anthropomorphization of God.

	Mean			
Response	Theists	Atheists	Difference	Correlation
Beliefs				
Satan exists.	.51	.02	.49***	.32***
Satan is God’s enemy.	.31	.33	-.02	.00
Satan intervenes in human affairs.	.63	.48	.15*	.07
Satan has a physical appearance.	.66	.79	-.13*	.05
Attributions				
Psychological properties	2.5	2.0	0.5*	.52***
Biological properties	0.7	0.5	0.2	.58***
Physical properties	1.2	0.8	0.4**	.60***
All properties	4.3	3.3	1.0**	.65***

*p < .05, **p < .01, ***p < .001
