# Supplementary material for: Theories of God: Explanatory coherence in religious cognition
Source: PLoS One. 2018 Dec 26;13(12):e0209758. doi: 10.1371/journal.pone.0209758 (PMC6306263; doi:10.1371/journal.pone.0209758)
Supplement: S4 Table — (PDF) [file pone.0209758.s004.pdf]

S4 Table. Responses to questions about Heaven and Hell by theists and atheists, plus correlations between responses and anthropomorphization of God.

	Mean			
Response	Theists	Atheists	Difference	Correlation
Heaven				
Heaven exists.	.80	.07	.73***	.34***
Heaven has a physical location.	.58	.62	-.04	.15*
Heaven has a physical appearance.	.69	.79	-.10	.17**
Human activities continue in Heaven.	.73	.69	.04	.19**
Hell				
Hell exists.	.54	.03	.51***	.39***
Hell has a physical location.	.54	.70	-.16**	.07
Hell has a physical appearance.	.73	.83	-.10	.09
Human activities continue in Hell.	.74	.83	-.09	.11

*p < .05, **p < .01, ***p < .001
