# Supplementary material for: Theories of God: Explanatory coherence in religious cognition
Source: PLoS One. 2018 Dec 26;13(12):e0209758. doi: 10.1371/journal.pone.0209758 (PMC6306263; doi:10.1371/journal.pone.0209758)
Supplement: S5 Table — (PDF) [file pone.0209758.s005.pdf]

S5 Table. Responses to questions about cosmogenesis and anthropogenesis by theists and atheists, plus correlations between responses and anthropomorphization of God.

	Mean			
Response	Theists	Atheists	Difference	Correlation
Cosmogenesis				
Universe was created by God.	.22	.01	.21***	.19**
Universe was created by the Big Bang.	.25	.83	-.58***	-.24***
Both explanations are correct.	.53	.17	.37***	.11
Anthropogenesis				
Humans were created by God.	.17	.01	.16***	.09
Humans evolved from other organisms.	.33	.97	-.63***	-.33***
Both explanations are correct.	.50	.03	.47***	.30***

*$p < .05$, **$p < .01$, ***$p < .001$
