# Supplementary material for: Theories of God: Explanatory coherence in religious cognition
Source: PLoS One. 2018 Dec 26;13(12):e0209758. doi: 10.1371/journal.pone.0209758 (PMC6306263; doi:10.1371/journal.pone.0209758)
Supplement: S6 Table — (PDF) [file pone.0209758.s006.pdf]

S6 Table. Responses to questions about suffering and misdeeds by theists and atheists, plus correlations between responses and anthropomorphization of God.

	Mean			
Response	Theists	Atheists	Difference	Correlation
Suffering				
God is not omnipotent.	.30	.80	-.50***	-.23***
God is not omnibenevolent.	.25	.74	-.49***	-.20**
God uses suffering to teach/punish.	.37	.08	.29***	.11
God allows suffering as part of larger plan.	.22	.05	.17***	.21***
Misdeeds				
God is not omniscient.	.17	.73	-.56***	-.30***
God does not judge humans.	.21	.72	-.51***	-.30***
God gave humans free will.	.39	.07	.32***	.19**
God wants humans to learn from mistakes.	.14	.06	.08*	.15*

*$p < .05$, **$p < .01$, ***$p < .001$
