# Supplementary material for: Theories of God: Explanatory coherence in religious cognition
Source: PLoS One. 2018 Dec 26;13(12):e0209758. doi: 10.1371/journal.pone.0209758 (PMC6306263; doi:10.1371/journal.pone.0209758)
Supplement: S7 Table — (PDF) [file pone.0209758.s007.pdf]

S7 Table. Responses to questions about religious practices, affiliation, and development by theists and atheists, plus correlations between responses and anthropomorphization of God.

	Mean			
Response	Theists	Atheists	Difference	Correlation
Prayer				
Prays occasionally	.73	.14	.59***	.24***
Prays daily	.25	.00	.25***	.22***
Prays in a ritualized fashion	.19	.03	.16***	.06
Prays for specific outcomes	.40	.07	.33***	.19**
Worship				
Attends religious services occasionally	.81	.48	.33***	.07
Attends religious services weekly	.26	.01	.25***	.22***
Religious development				
Acquired beliefs from a religious authority	.19	.12	.07	.05
Acquired beliefs from family and friends	.83	.76	.07	.04
Acquired beliefs from oneself	.21	.29	-.07	-.09
Had formal instruction in religion	.74	.52	.22***	.12*
Changed beliefs over time	.82	.74	.08	.03
Developed stronger/additional beliefs	.21	.04	.17***	.21***
Developed weaker/less dogmatic beliefs	.34	.57	-.22***	-.19**

Religious affiliation				
Protestant	.37	.11	.26***	.14*
Catholic	.28	.05	.23***	.04
Jewish	.12	.10	.02	-.06
Other (e.g., Hindu, Buddhist, Muslim)	.08	.10	-.01	.16**
Unaffiliated	.15	.64	-.49***	-.22***

*p < .05, **p < .01, ***p < .001
