# Supplementary material for: Theories of God: Explanatory coherence in religious cognition
Source: PLoS One. 2018 Dec 26;13(12):e0209758. doi: 10.1371/journal.pone.0209758 (PMC6306263; doi:10.1371/journal.pone.0209758)
Supplement: S8 Table — (PDF) [file pone.0209758.s008.pdf]

S8 Table. Correlations among psychological, biological, and physical properties attributions to God, angels, and Satan.

Measure	Being	Domain	1	2	3	4	5	6	7	8	9
1	God	Psychology	—	.43***	.62***	.58***	.31***	.37***	.65***	.37***	.46***
2		Biology		—	.82***	.24***	.48***	.44***	.26***	.61***	.51***
3		Physics			—	.37***	.44***	.48***	.38***	.55***	.58***
4	Angels	Psychology				—	.49***	.61***	.63***	.37***	.48***
5		Biology					—	.86***	.29***	.59***	.57***
6		Physics						—	.35***	.53***	.59***
7	Satan	Psychology							—	.45***	.62***
8		Biology								—	.79***
9		Physics									—

*$p < .05$, **$p < .01$, ***$p < .001$
